# Association between blood pressure control during aneurysm clipping and functional outcomes in patients with aneurysmal subarachnoid hemorrhage

**DOI:** 10.3389/fneur.2024.1415840

**Published:** 2024-05-27

**Authors:** Xiangning Han, Guogang Luo, Jiahao Li, Rui Liu, Ning Zhu, Shiliang Jiang, Wenlong Ma, Yawen Cheng, Fude Liu

**Affiliations:** Department of Neurology and Stroke Center, The First Affiliated Hospital of Xi'an Jiaotong University, Xi’an, Shaanxi, China

**Keywords:** subarachnoid hemorrhage, aneurysm, aortic blood pressure, variability, prognostic factors

## Abstract

**Objectives:**

We explored the relationship between blood pressure variability (BPV) during craniotomy aneurysm clipping and short-term prognosis in patients with aneurysmal subarachnoid hemorrhage to provide a new method to improve prognosis of these patients.

**Methods:**

We retrospectively analyzed the differences between patient groups with favorable modified Rankin Scale (mRS ≤ 2) and unfavorable (mRS > 2) prognosis, and examined the association between intraoperative BPV and short-term prognosis.

**Results:**

The intraoperative maximum systolic blood pressure (SBP_max_, *p* = 0.005) and the coefficient of variation of diastolic blood pressure (DBP_CV_, *p* = 0.029) were significantly higher in the favorable prognosis group. SBP_max_ (OR 0.88, 95%CI 0.80–0.98) and Neu% (OR 1.22, 95%CI 1.03–1.46) were independent influence factors on prognosis. Patients with higher standard deviations of SBP (82.7% vs. 56.7%; *p* = 0.030), DBP (82.7% vs. 56.7%; *p* = 0.030), and DBP_CV_ (82.7% vs. 56.7%; *p* = 0.030) had more favorable prognosis.

**Conclusion:**

Higher SBP_max_ (≤180 mmHg) during the clipping is an independent protective factor for a 90-day prognosis. These results highlight the importance of blood pressure (BP) control for improved prognosis; higher short-term BPV during clipping may be a precondition for a favorable prognosis.

## Introduction

Spontaneous subarachnoid hemorrhage (SAH) is the third most common type of stroke and is commonly associated with aneurysmal rupture (1). Globally, approximately 500,000 patients develop aneurysmal subarachnoid hemorrhage (aSAH) annually (2). Approximately a quarter of patients with SAH die before hospital admission; overall outcomes are improved in those admitted to hospitals; however, these survivors face years with a diminished quality of life (3). Age, hypertension, intraoperative and postoperative complications, surgical timing, and surgical methods are significant factors affecting the prognosis of aSAH (4). Conversely, the effect of blood pressure fluctuations on the prognosis of aSAH remains uncertain (5–7). Previous studies on blood pressure variability (BPV) have ignored the impact of surgical approaches and intraoperative blood pressure fluctuations on the prognosis of patients with aSAH. Consequently, the present study sought to examine the association between intraoperative BPV and 90-day prognosis in patients with aSAH who underwent craniotomy aneurysm clipping.

## Methods

### Study population

A total of 59 patients with aSAH were included in this retrospective study. All the patients underwent craniotomy aneurysm clipping at the Department of Neurosurgery of the First Affiliated Hospital of Xi’an Jiaotong University between January 2019 and December 2022. The inclusion criteria were as follows: (1) age 18–80 years; (2) spontaneous primary aSAH confirmed by CT scan and digital subtraction angiography (DSA); (3) admission within 72 h after symptom onset and aneurysm clipping within 36 h after admission; and (5) complete medical records. Patients with severe craniocerebral trauma, modified Rankin Scale (mRS) scores before onset exceeding 2, or hemodynamic instability were excluded. This study was reviewed and approved by the Ethics Committee of the First Affiliated Hospital of Xi’an Jiaotong University (No. XJTU1AF2023LSK-265). Written informed consent from the legal guardians of the participants was not required for this retrospective study in accordance with national and local guidelines.

### Clinical management

All patients underwent emergency clipping of ruptured intracranial aneurysms under general anesthesia with tracheal intubation. Blood pressure (BP) of patients obtained by invasive arterial pressure monitoring (patient monitor model: BeneView T8) through radial artery catheterization was recorded every 15 min from entering to leaving the operating room. Patients were transferred to the intensive care unit, and nimodipine was routinely administered to prevent cerebral vasospasm after surgery. Treatment was administered in strict accordance with the relevant clinical management guidelines.

### Data collection

The general data of patients were recorded as follows: (1) demographic characteristics (age and sex); (2) medical comorbidities (hypertension and diabetes); (3) personal history (smoking and drinking); (4) characteristics of the aneurysm (location, size, and number); (5) assessment of severity at admission [Glasgow Coma Scale, Hunt-Hess Scale, modified Fisher grade (m-Fisher), and World Federation of Neurosurgical Societies (WFNS) score]; and (6) laboratory results at admission [hemoglobin, white blood cell, platelet, neutrophil percentage (Neu%), aspartate aminotransferase, alanine aminotransferase, cholesterol, blood urea nitrogen, serum creatinine, and random blood glucose].

We calculated the following indexes of systolic blood pressure (SBP) and diastolic blood pressure (DBP) (8, 9): The mean, maximum (max), minimum (min), difference of maximum minus minimum (max–min), standard deviation (SD, SD= $\sqrt{\frac{1}{n-1}\overset{n-1}{\underset{i=1}{\sum }}{\left(X_{i}-X_{\mathrm{mean}}\right)}^{2}}$ ), coefficient of variation (CV, CV = (X_SD_/X_mean_) *100%) and successive variation (SV, SV= $\sqrt{\frac{1}{n-1}\overset{n-1}{\underset{i=1}{\sum }}{\left(X_{i+1}-X_{i}\right)}^{2}}$ ).

### Evaluation of prognosis

The prognosis was evaluated using the mRS at 90 days after onset. All patients were followed up by the same neurologist through telephone interviews. The mRS is a 7-point scale ranging from 0 (no symptoms) to 6 (death). The mRS was dichotomized, as previously published, into favorable (mRS ≤ 2) and unfavorable (mRS > 2) prognoses for the outcome analyses (10).

### Statistical analysis

All statistical analyses were performed using SPSS25.0 statistical package. Data were presented as mean ± standard deviation ( $\bar{x}$ ± s) for continuous symmetric distribution variables, median (M) and interquartile range (IQR) for continuous skewed distribution variables, and percentages for categorical variables. Differences between the two groups were assessed using univariate analysis with independent Student’s *t*-tests for continuous variables and the chi-squared test for categorical variables. For non-parametric variables, we used the Mann–Whitney U-test. The significant parameters in the univariate analysis were used as inputs in the multivariate binary logistic regression model for regression analysis. Spearman’s rank correlation was used for correlation analysis. In all the tests, *p* < 0.05 was considered statistically significant.

### Data availability

Data were recorded and stored in both paper and electronic databases. The supporting data for this study are available from the corresponding author on request.

## Results

Fifty-nine eligible patients were enrolled over the four-year interval, of whom 29 (49%) were male, and 30 (51%) were female. The average age was 57.0 ± 8.2 years. The clinical data of the 59 patients (41 in the favorable group and 18 in the unfavorable group) are summarized in Table 1. The aneurysm size (*p* = 0.025), Hunt–Hess grade (*p* = 0.006), m-Fisher grade (*p* = 0.010), WFNS grade (*p* = 0.001), Neu% (*p* = 0.004), and blood Glu (*p* = 0.013) at admission and the incidence of delayed cerebral ischemia (DCI, *p* = 0.007) were significantly higher in the unfavorable group (mRS > 2) than in the favorable group (mRS ≤ 2). The two groups showed no significant differences in the remaining clinical data.

**Table 1:** Comparison of clinical characteristics between patient groups with favorable and unfavorable prognosis.

Characteristic	Favorable prognosis 41 (69.5%)	Unfavorable prognosis 18 (30.5%)	*p* value
Age, year ( $\bar{x}$ ±s)	56.1 ± 7.7	58.9 ± 9.1	0.215
Sex, females (n, %)	18 (43.9%)	12 (66.7%)	0.107
Smoking (n, %)	15 (36.6%)	6 (33. 3%)	0.810
Drinking (n, %)	9 (22.0%)	8 (44.4%)	0.079
Hypertension (n, %)	26 (63.4%)	13 (72.2%)	0.511
Diabetes (n, %)	2 (4.9%)	2 (11.1%)	0.381
Aneurysm Location (n, %)			
Anterior circulation	37 (90.2%)	15 (83.3%)	0.644
Posterior circulation	4 (9.8%)	3 (16.7%)	
Aneurysm Number (n, %)			
Single	37 (90.2%)	15 (83.3%)	0.644
Multiple	4 (9.8%)	3 (16.7%)	
Aneurysm size (n, %)			
Small (*d* ≤ 0.5 cm)	22 (53.7%)	4 (22.2%)	**0.025**
Larger (*d* > 0.5 cm)	19 (46.3%)	14 (77.8%)	
Hunt-Hess score (n, %)			
I-II	17 (41.5%)	1 (5.6%)	**0.006**
III-V	24 (58.5%)	17 (94.4%)	
m-Fisher score (n, %)			
0–2	24 (58.5%)	4 (22.2%)	**0.010**
3–4	17 (41.5%)	14 (77.8%)	
WFNS classification (n, %)			
I-II	27 (65.9%)	3 (16.7%)	**0.001**
III-V	14 (34.1%)	15 (83.3%)	
DCI	10 (24.4%)	11 (61.1%)	**0.007**
Laboratory Results ( $\bar{X}$ ±S/M, Q)			
Hb, g/L	134.0 (120.5, 153.5)	135.0 (121.8, 150.8)	0.811
WBC, cells/L	11.1 (8.2, 13.1)	12.3 (8.6, 14.9)	0.505
PLT, cells/L	203.0 (162.5, 252.0)	187.5 (157.3, 241.0)	0.627
Neu, %	85.8 (78.2, 90.6)	91.3 (86.4, 94.5)	**0.004**
AST, U/L	21.0 (18.0, 29.0)	26.50 (21.5, 29.8)	0.106
ALT, U/L	25.0 (14.0, 29.5)	22.5 (15.3, 26.0)	0.479
CHO, mmol/L	4.5 ± 1.1	5.1 ± 1.2	0.055
BUN, mmol/L	4.8 ± 1.5	4.3 ± 1.6	0.299
Cr, μmol/L	45.0 (37.0, 57.5)	50.0 (32.8, 56.5)	0.941
Glu, mmol/L	6.81 (6.2, 7.8)	7.84 (7.2, 9.6)	**0.013**

m-Fisher, modified Fisher grade; WFNS, World Federation of Neurosurgical Societies score; DCI, delayed cerebral ischemia; Hb, hemoglobin; WBC, white blood cell; PLT, platelet; Neu%, neutrophil percentage; AST, aspartate aminotransferase; ALT, alanine aminotransferase; CHO, cholesterol; BUN, blood urea nitrogen; Cr, serum creatinine; Glu, random blood glucose.

Bold indicates statistical significance under the two-sided *p* < 0.05 assumption.

A comparison of the intraoperative SBP and DBP indices between the two groups is shown in Table 2. The SBP_max_ (152.2 ± 16.5 vs. 139.6 ± 12.7; *p* = 0.005) and DBPcv (10.8, IQR 8.5–12.8; 8.1, IQR 6.9–10.4; *p* = 0.029) were significantly higher in the favorable group than in the unfavorable group. Spearman’s correlation tests showed that intraoperative SBP_max_ (*r* = 0.34, *p* = 0.015) and DBPcv (*r* = 0.29, *p* = 0.027) were positively associated with a 90-day favorable prognosis.

**Table 2:** Comparison of intraoperative blood pressure parameters between patient groups with favorable and unfavorable prognosis.

Parameter (mmHg)	Favorable prognosis 41 (69.5%)	Unfavorable prognosis 18 (30.5%)	*p* value
SBP _mean_ ( $\bar{x}$ ±s)	122.3 ± 9.4	117.2 ± 9.3	0.058
SBP _max_ ( $\bar{x}$ ±s)	152.2 ± 16.5	139.6 ± 12.7	**0.005**
SBP _min_ (M, Q)	105.0 (98.0, 113.0)	100.0 (87.0, 109.3)	0.075
SBP _max-min_ ( $\bar{x}$ ±s)	46.4 ± 16.0	42.3 ± 19.3	0.395
SBP _SD_ ( $\bar{x}$ ±s)	11.3 ± 3.7	9.91 ± 2.9	0.160
SBP _CV_ ( $\bar{x}$ ±s)	9.3 ± 3.3	8.6 ± 2.9	0.401
SBP _SV_ (M, Q)	8.4 (7.9, 10.5)	9.9 (7.1, 14.4)	0.188
DBP _mean_ ( $\bar{x}$ ±s)	67.8 ± 7.0	68.3 ± 8.8	0.838
DBP _max_ ( $\bar{x}$ ±s)	88.2 ± 11.2	83.1 ± 12.5	0.132
DBP _min_ (M, Q)	57.0 (53.0, 61.5)	59.0 (50.5, 62.5)	0.921
DBP _max-min_ (M, Q)	29.0 (21.5, 39.0)	24.0 (18.3, 29.8)	0.106
DBP _SD_ (M, Q)	7.2 (5.6, 8.7)	5.71 (4.6, 7.2)	0.050
DBP _CV_ (M, Q)	10.8 (8.5, 12.8)	8.1 (6.9, 10.4)	**0.029**
DBP _SV_ (M, Q)	6.2 (5.1, 7.2)	6.6 (5.5, 8.7)	0.172

SBP, systolic blood pressure; DBP, diastolic blood pressure; max, maximum; min, minimum; max-min, difference of maximum minus minimum; SD, standard deviation; CV, coefficient of variation.

Bold indicates statistical significance under the two-sided *p* < 0.05 assumption.

**Figure 1:**
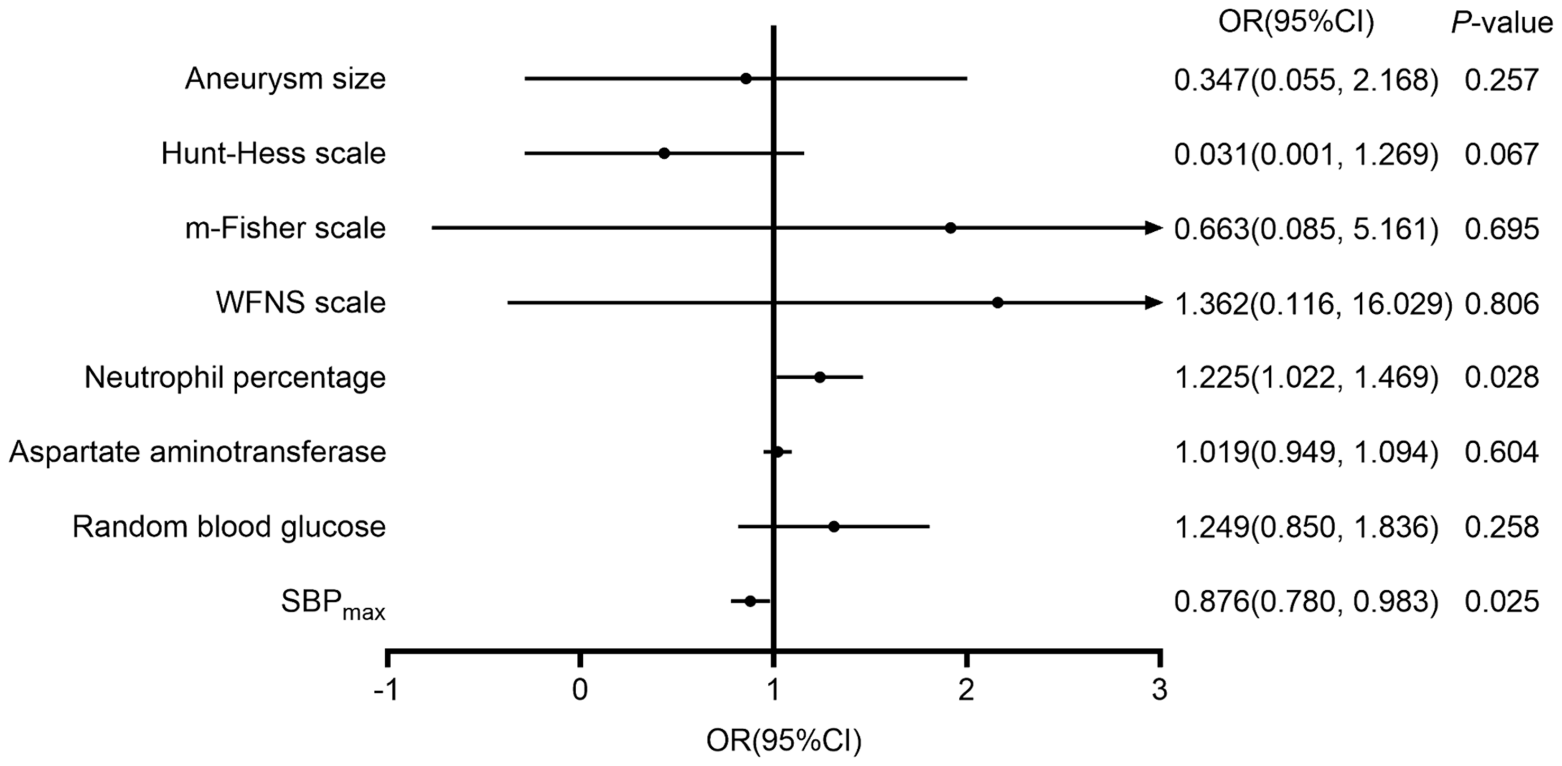
The influencing factors on the 90-day prognosis of patients with aneurysmal subarachnoid hemorrhage. m-Fisher, modified Fisher grade; WFNS, World Federation of Neurosurgical Societies score; SBP_max_, maximum systolic blood pressure; DBP_CV_, coefficient of variation of the diastolic blood pressure.

Indices with statistical differences in univariate analysis were included in the multivariate binary logistic regression analysis. This analysis showed that Neu% (OR 1.22, 95%CI 1.03–1.46) is an independent risk factor for unfavorable prognosis, while SBP_max_ (OR 0.88, 95%CI 0.80–0.98) is an independent protective factor for prognosis. Figure 1 shows the factors that significantly influenced the 90-day prognosis of patients with aSAH. In addition, we stratified the 47 patients with systolic BP ≤180 mmHg and the 56 patients with ≤160 mmHg, according to the 95^th^ and 90th percentiles, respectively. The results showed that when the SBP_max_ was ≤180 mmHg, the probability of developing a favorable prognosis at 90 days increased with increasing SBP_max_ (OR 1.06, 95% CI 1.01–1.11; *p* = 0.018). However, the trend was not statistically significant when the cutoff value was 160 mmHg (OR 1.04, 95% CI 0.98–1.10; *p* = 0.212).

**Figure 2:**
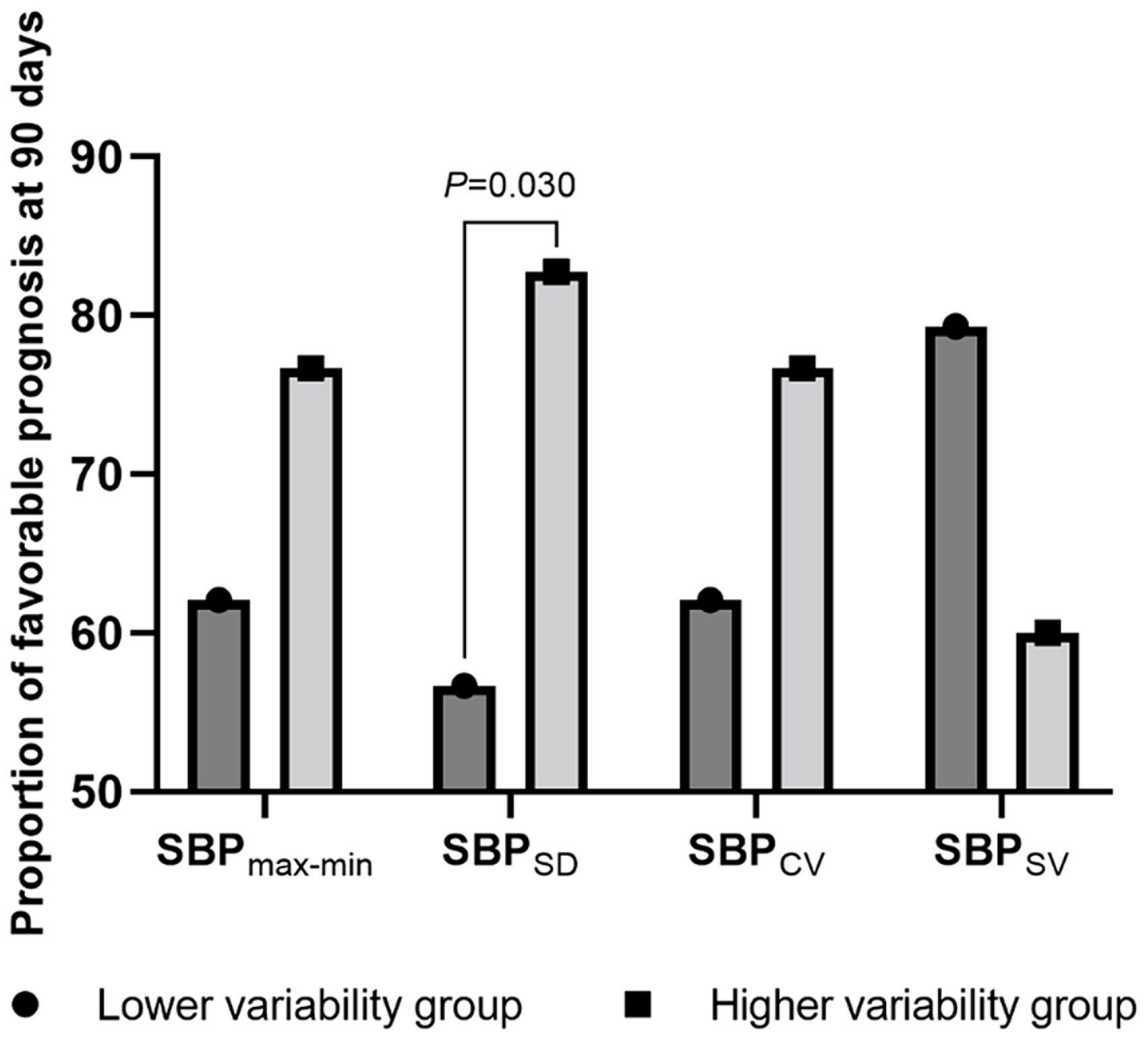
The proportion of favorable prognosis at 90 days for patient groups with lower and higher systolic blood pressure variability. SBP, systolic blood pressure; max-min, difference of maximum minus minimum; SD, standard deviation; CV, coefficient of variation; SV, successive variation.

**Figure 3:**
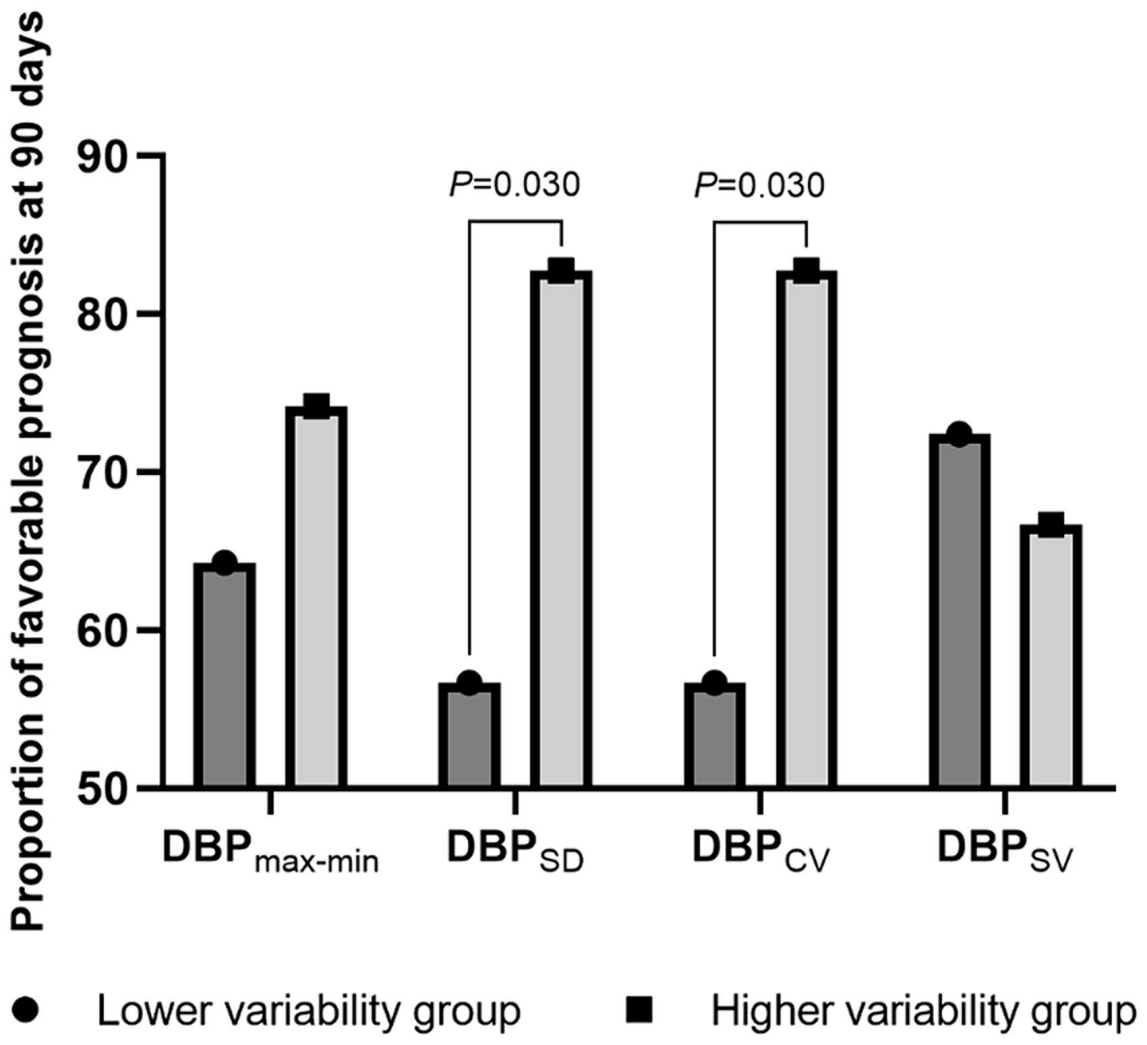
The proportion of favorable prognosis at 90 days for patient groups with lower and higher diastolic blood pressure variability during surgery. DBP, diastolic blood pressure; max-min, difference of maximum minus minimum; SD, standard deviation; CV, coefficient of variation; SV, successive variation.

Next, we stratified the patients into two groups based on the median BP variability parameters and compared their proportions of favorable prognoses. Patients with a higher SBP_SD_ (56.7% vs. 82.7%; *p* = 0.030), DBP_SD_ (56.7% vs. 82.7%; *p* = 0.030), and DBP_CV_ (56.7% vs. 82.7%; *p* = 0.030) had a more favorable prognosis. Detailed data are presented in Figures 2, 3.

## Discussion

Our study demonstrated that intraoperative SBP_max_ is an independent predictor of favorable outcomes in patients with aSAH. Acute hypertension should be managed after developing aSAH; however, the parameters for appropriate BP control have not yet been defined. Insufficient management of BP may increase the risk of rebleeding, whereas aggressive treatment of hypertension increases the risk of ischemic stroke (11). It has been confirmed that induced hypertension can reduce the occurrence of DCI; however, unsurprisingly, having an SBP_max_ > 180 mmHg increases the risk of rebleeding (12, 13). In 2012, the AHA/ASA issued guidelines for SAH treatments, pointing out that maintaining euvolemia and inducing hypertension are effective measures to prevent DCI and recommended that SBP should be brought below 160 mmHg before clipping the aneurysm to reduce the risk of rebleeding (14). In 2013, the European Stroke Organization suggested that SBP should be maintained below 180 mmHg before surgery (13). In a previous study, Ascanio et al. reported that hypotension was independently associated with poor outcomes in patients with aSAH (15). Similarly, the present study showed that intraoperative SBP_max_ was an independent protective factor for patients with aSAH when it was below 180 mmHg. However, no protective effect was observed when SBP_max_ was <160 mmHg. These results led us to propose that the intraoperative SBP_max_ should be elevated but not exceed 180 mmHg, which is consistent with the management of preoperative hypertension proposed by the European Stroke Organization in 2013 (13). In addition, intraoperative hypotension appears to promote DCI and poor prognosis in patients with aSAH; however, the downline of intraoperative blood pressure control is inconsistent (16–19).

The unfavorable prognosis of patients with aSAH is often related to DCI, which is generally attributed to abnormal constriction of the cerebral arteries, namely, cerebral vasospasm (CVS) (20). DCI is associated with the autoregulatory failure of the nervous system (21). Furthermore, intracranial pressure variability (ICPV) is mainly affected by fluctuations in cerebral blood flow, which indirectly reflects cerebral autoregulation (22). Previous studies have shown that low BPV and ICPV are associated with unfavorable outcomes in patients with aSAH (7). The present results show that patients with higher BP variability have a higher incidence of favorable prognoses. In 1929, Walter B. Cannon first proposed the concept of “homeostasis.” According to self-regulation mechanisms, physiological homeostasis is not static but maintains a dynamic equilibrium within a tolerable range. Physiological variability reflects the ability of an organism to autoregulate. Abnormal variability, reflecting impaired physiological responsiveness, can increase the risk of further physiological derangement (23). DBP is mainly affected by the heart rate and peripheral resistance. After SAH, the concentration of calcium ions in the cerebrospinal fluid increases, and the flow of calcium ions into the cells accelerates, resulting in vasoconstriction and increased vascular resistance. A lower DBP_CV_ in patients with aSAH implies distal microvascular spasms, indicating an increased risk of CVS or even DCI, which is closely related to unfavorable prognoses. However, other studies have shown that variability in SBP within 24 h of admission is an independent risk factor for poor prognosis in patients with SAH (6). These propositions were not entirely consistent, possibly owing to inconsistencies arising from the different timeframes considered in the variability assessment. Higher short-term variability may reflect faster and more effective functioning of adaptive mechanisms, and higher long-term variability (time spans exceeding 1 h) may reflect a decreased integrated ability of adaptive mechanisms to respond to challenges (7). Therefore, the timescale of BP variability determines its effect on clinical outcomes.

Previous studies have shown that BP variability is related to the vegetative nervous system function, such that decreased variability implies autonomic nerve dysfunction (24, 25). If the sympathetic nervous system is overactivated, feedback regulation of the neuroendocrine system may fail, leading to decreased short-term variability and an increased risk of unfavorable prognoses (26). Supporting this model, pharmacological blocking of the cervical sympathetic ganglion can effectively relieve DCI after SAH (27), which has also been confirmed in animal experiments (28). As mentioned above, timely and effective inhibition of sympathetic hyperactivity may benefit patients with aSAH, offering a new therapeutic target to reduce the incidence of CVS and improve prognosis.

The small sample size used during this study may have reduced the generalizability of the results; however, the overall trend was compelling. The mean duration of intracranial aneurysm clipping among the enrolled patients was only 5.4 h, so we could not analyze longer-term BPV. Furthermore, not all BP values were available during the entire hospital stay. Therefore, we cannot discuss the prognostic effects of BPV during hospitalization. We did not fully consider the influence of intraoperative drugs on BPV, which may have affected the accuracy of the research findings and should, therefore, be considered in future-related studies. We intend to conduct further clinical studies to evaluate whether patients benefit from intraoperative interventions for arterial pressure reduction.

In conclusion, SBP_max_ during craniotomy aneurysm clipping was an independent protective factor for a favorable prognosis at 90 days. Appropriately high BP is beneficial for improving prognosis. However, the risk of adverse effects such as rebleeding should be considered. A higher short-term BPV during surgery implies higher cerebral autoregulatory function, potentially leading to a more favorable prognosis.

## Data availability statement

The raw data supporting the conclusions of this article will be made available by the authors, without undue reservation.

## Ethics statement

The studies involving humans were approved by the Ethics Committee of the First Affiliated Hospital of Xi'an Jiaotong University. The studies were conducted in accordance with the local legislation and institutional requirements. The ethics committee/institutional review board waived the requirement of written informed consent for participation from the participants or the participants’ legal guardians/next of kin due to the retrospective nature of the study.

## Author contributions

XH: Writing – original draft, Methodology, Data curation, Conceptualization. GL: Writing – review & editing, Supervision, Resources, Project administration. JL: Writing – review & editing, Visualization, Investigation. RL: Writing – review & editing, Investigation. NZ: Writing – review & editing, Investigation. SJ: Writing – review & editing, Data curation. WM: Writing – review & editing, Data curation. YC: Writing – review & editing, Methodology, Funding acquisition, Conceptualization. FL: Writing – review & editing, Methodology, Funding acquisition, Conceptualization.

## References

[1] Feigin VL Lawes CM Bennett DA Barker-Collo SL Parag V. Worldwide stroke incidence and early case fatality reported in 56 population-based studies: a systematic review. Lancet Neurol. (2009) 8:355–69. doi: 10.1016/S1474-4422(09)70025-0, PMID: 19233729

[2] Hughes JD Bond KM Mekary RA Dewan MC Rattani A Baticulon R. Estimating the global incidence of aneurysmal subarachnoid hemorrhage: a systematic review for central nervous system vascular lesions and meta-analysis of ruptured aneurysms. World Neurosurg. (2018) 115:430–447.e7. doi: 10.1016/j.wneu.2018.03.220, PMID: 29649643

[3] Claassen J Park S. Spontaneous subarachnoid haemorrhage. Lancet. (2022) 400:846–62. doi: 10.1016/S0140-6736(22)00938-2, PMID: 35985353 PMC9987649

[4] Ge XB Yang QF Liu ZB Zhang T Liang C. Increased blood pressure variability predicts poor outcomes from endovascular treatment for aneurysmal subarachnoid hemorrhage. Arq Neuropsiquiatr. (2021) 79:759–65. doi: 10.1590/0004-282x-anp-2020-0167, PMID: 34669812

[5] Chung P-W Kim J-T Sanossian N Starkmann S Hamilton S Gornbein J. Association between hyperacute stage blood pressure variability and outcome in patients with spontaneous intracerebral hemorrhage. Stroke. (2018) 49:348–54. doi: 10.1161/STROKEAHA.117.017701, PMID: 29301973

[6] Yang M Pan X Liang Z Huang X Duan M Cai H. Association between blood pressure variability and the short-term outcome in patients with acute spontaneous subarachnoid hemorrhage. Hypertens Res. (2019) 42:1701–7. doi: 10.1038/s41440-019-0274-y, PMID: 31171841

[7] Kirkness CJ Burr RL Mitchell PH. Intracranial and blood pressure variability and long-term outcome after aneurysmal sub-arachnoid hemorrhage. Am J Crit Care. (2009) 18:241–51. doi: 10.4037/ajcc2009743, PMID: 19411584 PMC2754727

[8] Rothwell PM Howard SC Dolan E O'Brien E Dobson JE Dahlöf B. Prognostic significance of visit-to-visit variability, maximum systolic blood pressure, and episodic hypertension. Lancet. (2010) 375:895–905. doi: 10.1016/S0140-6736(10)60308-X, PMID: 20226988

[9] Schachinger H Langewitz W Schmieder RE Ruddel H. Comparison of paramaters for assessing blood-pressure and heart-rate variability from non-invasive 24-hour blood pressure monitoring. J Hypertens. (1989) 7:S81–4. 2760718

[10] Berkhemer OA Fransen PSS Beumer D Van Den Berg LA Lingsma HF Yoo AJ. A randomized trial of intraarterial treatment for acute ischemic stroke. N Engl J Med. (2015) 372:11–20. doi: 10.1056/NEJMoa1411587 25517348

[11] Wijdicks EFM Vermeulen M Murray GD Hijdra A Vangijn J. The effects of treating hypertension following aneurysmal subarachnoid hemorrhage. Clin Neurol Neurosurg. (1990) 92:111–7. doi: 10.1016/0303-8467(90)90085-J 2163791

[12] Haegens NM Gathier CS Horn J Coert BA Verbaan D Van Den Bergh WM. Induced hypertension in preventing cerebral infarction in delayed cerebral ischemia after subarachnoid hemorrhage. Stroke. (2018) 49:2630–6. doi: 10.1161/STROKEAHA.118.022310 30355184

[13] Steiner T Juvela S Unterberg A Jung C Forsting M Rinkel G. European stroke organization guidelines for the management of intracranial aneurysms and subarachnoid haemorrhage. Cerebrovasc Dis. (2013) 35:93–112. doi: 10.1159/000346087, PMID: 23406828

[14] Connolly ES Jr Rabinstein AA Carhuapoma JR Derdeyn CP Dion J Higashida RT. Guidelines for the management of aneurysmal subarachnoid hemorrhage a guideline for healthcare professionals from the American Heart Association/American Stroke Association. Stroke. (2012) 43:1711–37. doi: 10.1161/STR.0b013e3182587839 22556195

[15] Ascanio LC Enriquez-Marulanda A Maragkos GA Salem MM Alturki AY Ravindran K. Effect of blood pressure variability during the acute period of subarachnoid hemorrhage on functional outcomes. Neurosurgery. (2020) 87:779–87. doi: 10.1093/neuros/nyaa019, PMID: 32078677

[16] Wang J Li R Li S Ma T Zhang X Ren Y. Intraoperative arterial pressure and delayed cerebral ischemia in patients with aneurysmal subarachnoid hemorrhage after surgical clipping: a retrospective cohort study. Front Neurosci. (2023) 17:1064987. doi: 10.3389/fnins.2023.1064987, PMID: 36875639 PMC9982002

[17] Hoff RG VaND G Mettes S Verweij BH Algra A Rinkel GJ. Hypotension in anaesthetized patients during aneurysm clipping: not as bad as expected? Acta Anaesthesiol Scand. (2008) 52:1006–11. doi: 10.1111/j.1399-6576.2008.01682.x, PMID: 18494846

[18] Chang HS Hongo K Nakagawa H. Adverse effects of limited hypotensive anesthesia on the outcome of patients with subarachnoid hemorrhage. J Neurosurg. (2000) 92:971–5. doi: 10.3171/jns.2000.92.6.0971, PMID: 10839257

[19] Chong JY Kim DW Jwa CS Yi HJ Ko Y Kim KM. Impact of cardio-pulmonary and intraoperative factors on occurrence of cerebral infarction after early surgical repair of the ruptured cerebral aneurysms. J Korean Neurosurg Soc. (2008) 43:90–6. doi: 10.3340/jkns.2008.43.2.90, PMID: 19096611 PMC2588225

[20] Rowland MJ Hadjipavlou G Kelly M Westbrook J Pattinson KTS. Delayed cerebral ischaemia after subarachnoid haemorrhage: looking beyond vasospasm. Br J Anaesth. (2012) 109:315–29. doi: 10.1093/bja/aes264, PMID: 22879655

[21] Budohoski KP Czosnyka M Kirkpatrick PJ Smielewski P Steiner LA Pickard JD. Clinical relevance of cerebral autoregulation following subarachnoid haemorrhage. Nat Rev Neurol. (2013) 9:152–63. doi: 10.1038/nrneurol.2013.11, PMID: 23419369

[22] Svedung Wettervik T Howells T Hanell A Ronne-Engstrom E Lewen A Enblad P. Low intracranial pressure variability is associated with delayed cerebral ischemia and unfavorable outcome in aneurysmal subarachnoid hemorrhage. J Clin Monit Comput. (2021) 36:569–78. doi: 10.1007/s10877-021-00688-y 33728586 PMC9123038

[23] Goldstein B Fiser DH Kelly MM Mickelsen D Ruttimann U Pollack MM. Decomplexification in critical illness and injury: relationship between heart rate variability, severity of illness, and outcome. Crit Care Med. (1998) 26:352–7. doi: 10.1097/00003246-199802000-00040, PMID: 9468175

[24] Soehle M Czosnyka M Chatfield DA Hoeft A Pena A. Variability and fractal analysis of middle cerebral artery blood flow velocity and arterial blood pressure in subarachnoid hemorrhage. J Cereb Blood Flow Metab. (2008) 28:64–73. doi: 10.1038/sj.jcbfm.9600506 17473850

[25] Schmidt JM. Heart rate variability for the early detection of delayed cerebral ischemia. J Clin Neurophysiol. (2016) 33:268–74. doi: 10.1097/WNP.0000000000000286, PMID: 27258451

[26] Constantinescu V Matei D Ignat B Hodorog D Cuciureanu DI. Heart rate variability analysis a useful tool to assess poststroke cardiac dysautonomia. Neurologist. (2020) 25:49–54. doi: 10.1097/NRL.0000000000000270 32358461

[27] Treggiari MM Romand JA Martin JB Reverdin A Rufenacht DA De Tribolet N. Cervical sympathetic block to reverse delayed ischemic neurological deficits after aneurysmal subarachnoid hemorrhage. Stroke. (2003) 34:961–7. doi: 10.1161/01.STR.0000060893.72098.80, PMID: 12649526

[28] Hu N Wu Y Chen BZ Han JF Zhou MT. Protective effect of stellate ganglion block on delayed cerebral vasospasm in an experimental rat model of subarachnoid hemorrhage. Brain Res. (2014) 1585:63–71. doi: 10.1016/j.brainres.2014.08.012 25128600

